# Early national trends in non-abortion reproductive care access after *Roe*

**DOI:** 10.3389/fpubh.2024.1309068

**Published:** 2024-03-08

**Authors:** Junying Zhao, Aaron Zahn, Samuel C. Pang, Tony S. Quang, Janis Campbell, Perry N. Halkitis

**Affiliations:** ^1^Department of Health Administration and Policy, University of Oklahoma Health Sciences Center, Oklahoma City, OK, United States; ^2^The Lexington Center IVF Inc, Boston, MA, United States; ^3^Department of Radiation Oncology, Tibor Rubin VA Medical Center, Long Beach, CA, United States; ^4^Department of Biostatistics and Epidemiology, University of Oklahoma Health Sciences Center, Oklahoma City, OK, United States; ^5^Department of Biostatistics and Epidemiology, Rutgers University, Piscataway, NJ, United States

**Keywords:** *Roe* v. Wade, *Dobbs* v. Jackson Women’s Health Organization, reproductive health services, abortion clinics, Title X entities, artificial reproductive technology clinics, access, equity

## Abstract

**Background:**

*Roe* was overturned in 2022. No peer-reviewed evidence exists for the indirect spillover effects of overturning *Roe* on non-abortion reproductive care access for diverse patient populations.

**Methods:**

National data were from 2013–2023 HHS Title X Directory, 2013–2020 CDC Artificial Reproductive Technologies (ART) Surveillance and 2021–2023 manual collection, and Guttmacher Institute. Outcome measures included numbers of ART clinics and Title X entities. Title X entities are those that receive federal funds to establish and operate voluntary family planning projects, especially for low-income patients. We reported pre-and post-*Roe* changes, associations between changes in measures and abortions, and characteristics of changed measures by region and political geography.

**Results:**

Post-*Roe* America witnessed national declines of 1.03% in ART clinics and 18.34% in Title X entities, and average state decreases of 0.08 ART clinics (*p* < 0.05) and 18 Title X entities (*p* < 0.001). State-level ART clinic closures and abortion reductions had little association except for Texas, Oklahoma, Arizona, New York, and California. Plummets in Title X entities and abortions were positively associated: Reducing 100 abortions was associated with defunding two Title X entities (*p* < 0.05). The South experienced the largest losses of both, while 83.39% of lost Title X entities were in states that voted Republican in the 2020 presidential election, disproportionate to the 49.02% of states that voted Republican and the 42.52% of US population residing in these states.

**Conclusion:**

We provide one of the first few evidence of spillover impacts of overturning *Roe* on non-abortion care access for diverse populations: low-income men and women, single parents by choice, and biologically and socially infertile patients. Early evidence warns of worsening challenges of inequities and calls for immediate policy actions.

## Introduction

Procreation is a fundamental right protected by the Fourteenth Amendment to the US Constitution (1), along with other essential reproductive rights (2). Historical state statutes deprived certain individuals of the right to have children and were challenged. In *Skinner v. Oklahoma (1942)*, the US Supreme Court struck down compulsory sterilization laws and affirmed that “procreation [is] fundamental to the very existence and survival of the race” (3).

However, recent US Supreme Court rulings may prevent diverse populations from exercising the right to procreate. The Court accepted *Dobbs v. Jackson Women’s Health Organization (2022)* for review on May 17, 2021. The issue was “whether all pre-viability prohibitions on elective abortions are unconstitutional.” The Court held that “the Constitution does not confer a right to abortion; *Roe* and *Casey* are overruled” on June 24, 2022 (4). The decision triggered immediate enactment of statutes in 13 states that enforced near-total bans on abortion (5). In response, the National Academy of Medicine emphasized potential consequences on health inequities among women (6). The US Congress held hearings on post-*Roe* abortion policy (7), and the Biden Administration issued an executive order protecting access to reproductive care in July 2022 (8). The direct effects of overturning *Roe* on reduced abortion access have been discussed and empirically documented (9).

No peer-reviewed study has quantified the indirect spillover impacts of overturning *Roe* on non-abortion care access for diverse patient populations. Broad-spectrum reproductive services include not only abortion but also contraceptive, fertility, preventive, maternal and prenatal health services (2). Diverse stakeholders stressed the potential spillover impacts on these services (10, 11). The American Society for Reproductive Medicine declared: “The clearest danger is the ambiguity about the legal status of *in vitro* fertilized [IVF] eggs” (12). Indeed, some abortion trigger laws define an unborn child as an embryo at any gestational stage from fertilization to birth. Such restrictive legal definitions of an embryo as a person may discourage assisted reproductive technology (ART) service supplies, harming patient access (5). Yet, no relevant evidence has existed about the spillover impacts of overturning *Roe* on non-abortion reproductive service supply, thus access. An urgent need presents to disentangle the complexity of post-*Roe* reproductive care access from multiple sources: the relationships between abortion and non-abortion care, federal and state health authorities, and judicial and legislative checks and balances.

This article is the first that used administrative and manually-collected data to report post-*Roe* early national trends of declining non-abortion care access, and their positive associations with diminishing abortions in certain states and the nation. It documents early evidence on the spillover impacts of federal judicial overturn of abortion precedent on nationwide state-level non-abortion care access through state abortion trigger laws as mechanisms. As gender, income, marital status, and sexual orientation inequities endure in non-abortion care access for diverse patient populations, the early evidence warns of worsening challenges and calls for immediate policy actions.

## Measures and data

We extracted nationwide state-level administrative data, whose current data were partially unreleased and thus manually collected, and compared two measures of non-abortion reproductive care access before and after the overturn of *Roe*. Forward-looking agents (e.g., administrators and physicians) make decisions based on predictions (13). An overturn was predicted as more likely (14). Thus, we also compared measures before and after the review of *Dobbs*.

The two measures were the numbers of ART clinics and entities that receive funding through Title X of the US Public Health Service Act (PHSA). They largely complement each other regarding the tax-exempt status of organization, type of provided services, service insurance coverage, and patient income levels. Title X entities are public or nonprofit private entities that receive federal funds to establish and operate voluntary family planning projects, especially for low-income patients. The PHSA does not define “voluntary family planning projects,” which commonly include contraceptive and preventive services, does not explicitly include or exclude advanced fertility services (e.g., ART), but does explicitly exclude abortion as a reimbursable service (15).

In contrast, ART clinics are usually for-profit private entities (16), and procedures are often expensive and uncovered by insurance. In the US in 2017, an IVF cycle cost about $12,400 (17), and only 26% of employers with over 500 employees included IVF in employer-sponsored insurance plans (18). Unsurprisingly, we found in 2020 data that no ART clinics received Title X funding. The numbers of ART clinics and Title X entities complementarily measure access to non-abortion reproductive services.

Administrative data were extracted from the US Department of Health and Human Services (HHS) Title X Family Planning 2013–2021 Annual Reports and 2022–2023 Monthly Directory (19), and the Center for Disease Control and Prevention (CDC) 2013–2020 Annual ART Fertility Clinic and National Summary Reports. The CDC takes two years to process and release such data (20). As 2023 data will be unavailable until 2025, we collected the 2021–2023 operation status of all 495 clinics, and closure date if applicable, in the 2020 report manually.

In addition, we sought to detect whether changes in ART clinics or Title X entities were associated with changes in abortion clinics. Recall that the primary objective of ART clinic services is to induce pregnancies, while that of abortion clinic services is to terminate pregnancies. Thus, the number of ART services and clinics and that of abortion services and clinics are seemingly uncorrelated. However, consistent with the hypothesis in literature (11), we suspect that these two numbers are logically correlated as a result of the federal judicial law change. Specifically, the federal judicial overturn of the abortion precedent may negatively impact ART clinics through the mechanism of state abortion trigger laws that restrict the definition of personhood of embryos; that is, changes in ART and abortion clinics may be positively associated. Similarly, we also suspect that changes in Title X entities and abortion clinics are positively associated. This hypothesis is motivated by the following two facts. First, a Title X entity may refer patients to an abortion clinic upon request. Second, an entity can simultaneously provide non-abortion services using Title X funds and provide abortion services for which Title X funds are prohibited (15). For example, Planned Parenthood treated about 40% of 1.7 million Title X patients (21) and conducted over 383,000 abortions in 2021 (22). Therefore, either through the complementary referral relationship between Title X entities and abortion clinics or through the complementary services relationship within the same entity, the changes in Title X and abortion entities may also be positively associated.

Data on changes in abortion clinics were publicly unavailable from the CDC Abortion Surveillance Report (23) and other administrative sources after 2020 and incomplete from the Guttmacher Institute (24), which would reduce the statistical power and preclude unbiased estimates. Therefore, we use a proxy measure, changes in abortions performed in each of the 50 states and DC, whose complete data were available during April–December 2022 from the Society of Family Planning (25) and Guttmacher Institute; these are the only complete and publicly available data as of this writing.

For each measure, national and state-level changes before (May 2022) and after (February 2023) the overturn were reported. We also reported the descriptive results of Pearson correlation and association between changes in ART clinics and abortions (April–December 2022), similarly for Title X entities. Finally, we reported characteristics of changed clinics or entities from the review (May 18, 2021 or April 2021) to date (February 2023) and from the overturn (June 25, 2022 or May 2022) to date by census region, political geography, and publicly-released reason for closure. April 2021 and May 2022 were chosen as bases to calculate changes in Title X entities, rather than May 2021 and June 2022, in which the review and overturn occurred because the data were monthly and changes may have already occurred in the remaining dates of these months.

## Early national trends based on post-*Roe* data

### ART clinics before-after overturn

**Figure 1:**
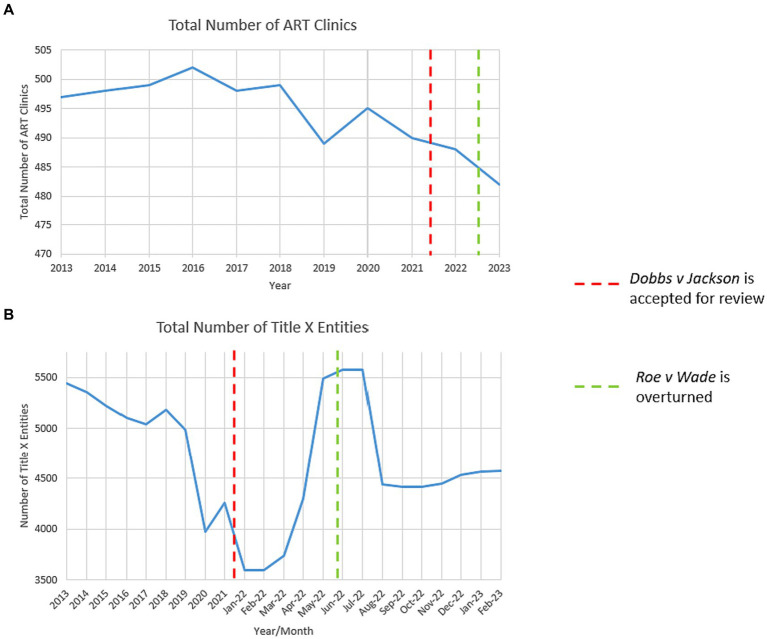
2013–2023 national trends in assisted reproductive technology (ART) clinics and Title X entities. **(A)** The total number of ART clinics in the US from 2013 to 2023. Data were retrieved from the HHS-CDC 2013–2020 annual ART Fertility Clinic and National Summary Reports (20). All clinics reporting and unreporting success data were included in this graph. The 2021–2023 annual reports are unavailable as of this writing; we manually collected operation status data of all 495 clinics in the 2020 report and, if closed, the closure date. We estimated 2021 ART clinics by subtracting closed ones from the 2020 report and estimated 2022 and 2023 ART clinics similarly. We observed a drop in the total number of ART clinics in 2020 at the onset of the COVID-19 pandemic, a fall in 2021 when *Dobbs* was accepted for review, and a steeper decrease in 2022 when *Roe* was overturned. **(B)** The total number of Title X entities (grantees, sub-recipients, and service sites) in the US from 2013 to 2023. Data were retrieved from the HHS-Office of Population Affairs (OPA) 2013–2021 Family Planning Annual Reports (19). The 2022 and 2023 annual reports are unavailable as of this writing, and we retrieved monthly data from the OPA Title X Directory from January 2022 to February 2023 (19). We observed a sharp drop in 2019 when the March 2019 Trump gag rule took effect, a surge in January 2022 following the November Biden-Harris rule repealing the gag rule, and shortly after the overturn in June 2022, a plummet starting in July 2022.

**Figure 2:**
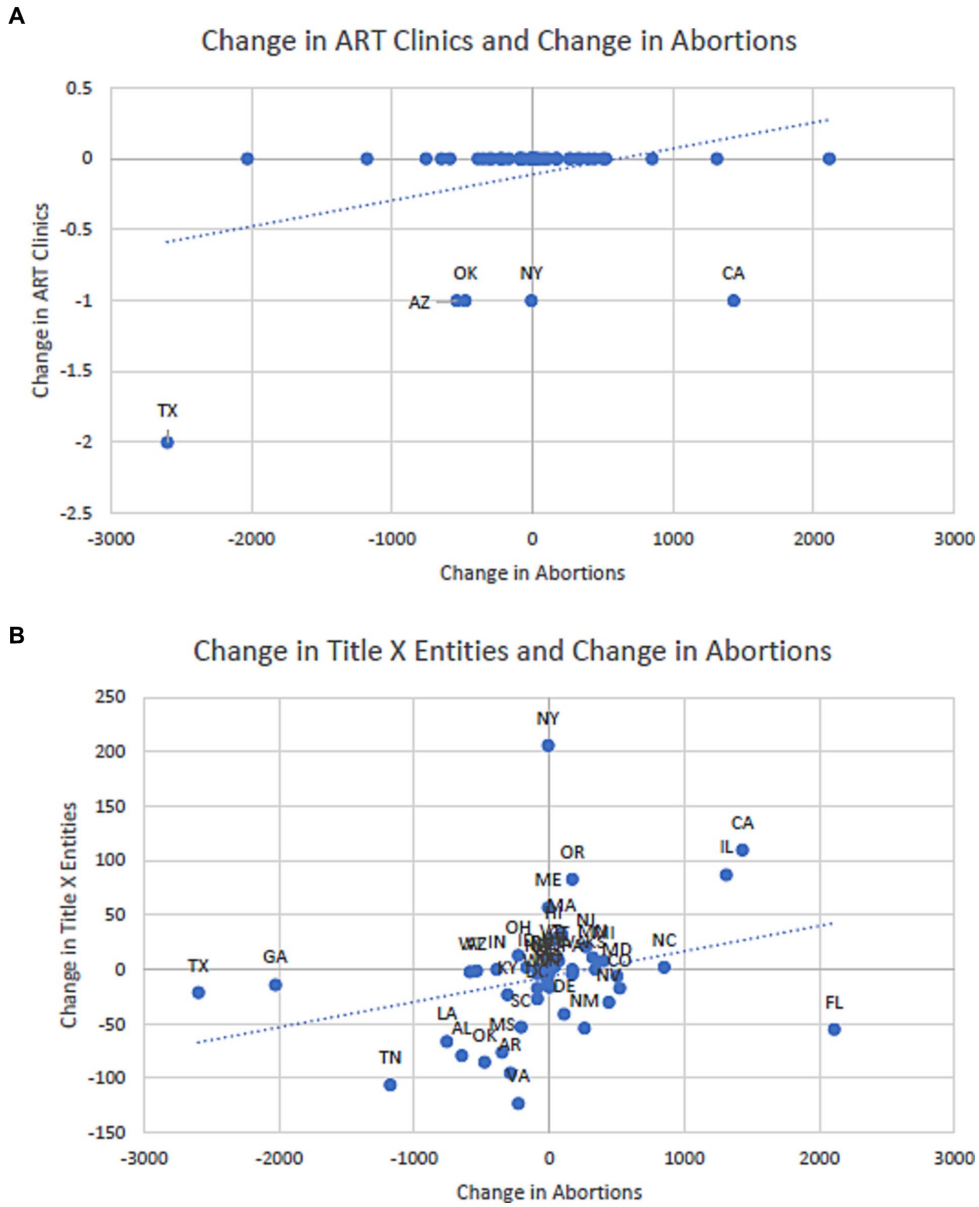
Correlation and association between pre-post *Roe* changes in ART clinics or Title X entities and changes in abortions. **(A)** Changes in ART clinics and abortions, April–December, 2022. **(B)** Changes in Title X entities and abortions, April–December, 2022. **(A)** Little correlation and association between changes in ART clinics and changes in abortions carried out in all 50 states and DC from April to December 2022, except for five outliers: Texas, Oklahoma, Arizona, and New York, with positive correlations and California with a negative correlation. Data on abortions were retrieved from the Society of Family Planning (25), which were further from the Guttmacher Institute. **(B)** The positive correlation and association between changes in Title X entities and changes in abortions carried out in all 50 states and DC from April to December 2022. The correlation [*r*(49) = 0.304; *p* = 0.029] and association were statistically significant (*β* = 0.023; 95% CI, 0.003 to 0.044): 100 abortions reduced in a state were associated with approximately two Title X entities being defunded.

A national declining trend existed in ART clinics in the past decade (Figure 1A). Four ART clinics were closed between March 2020 and March 2021, two due to mergers and two due to the COVID-19 pandemic. ART clinics dropped from 487 before the overturn to 482 to date, a 1.03% decrease nationwide. State-level group means before and after the overturn indicated a statistically significant average closure of 0.08 ART clinics per state (95% confidence interval [CI], −0.0021 to −0.1579; *p* = 0.022). Moreover, state-level changes in ART clinics and abortions show little association except for Texas, Oklahoma, Arizona, and New York, with positive relationships, and California, with a negative relationship (Figure 2A).

### Characteristics of closed ART clinics after review (May 18, 2021)

From the review of *Dobbs* to date, eight ART clinics have closed. Regarding regions, five (62.5%) were located in the South, two (25%) in the West, one (12.5%) in the Northeast, and none in the Midwest. Concerning political geography, six (75%) were in precincts that voted Democratic in the 2020 US presidential election, and two (25%) in precincts that voted Republican (26). Four were in states that voted Democratic, and four were in those that voted Republican. Closure reasons varied. Three clinics (37.5%) were closed because of practice cessation, two (25%) because of relocation, merger, or acquisition, one (12.5%) because of financial losses, and two (25%) with unspecified reasons (see Supplementary material for references).

### Characteristics of closed ART clinics after overturn (June 25, 2022)

**Figure 3:**
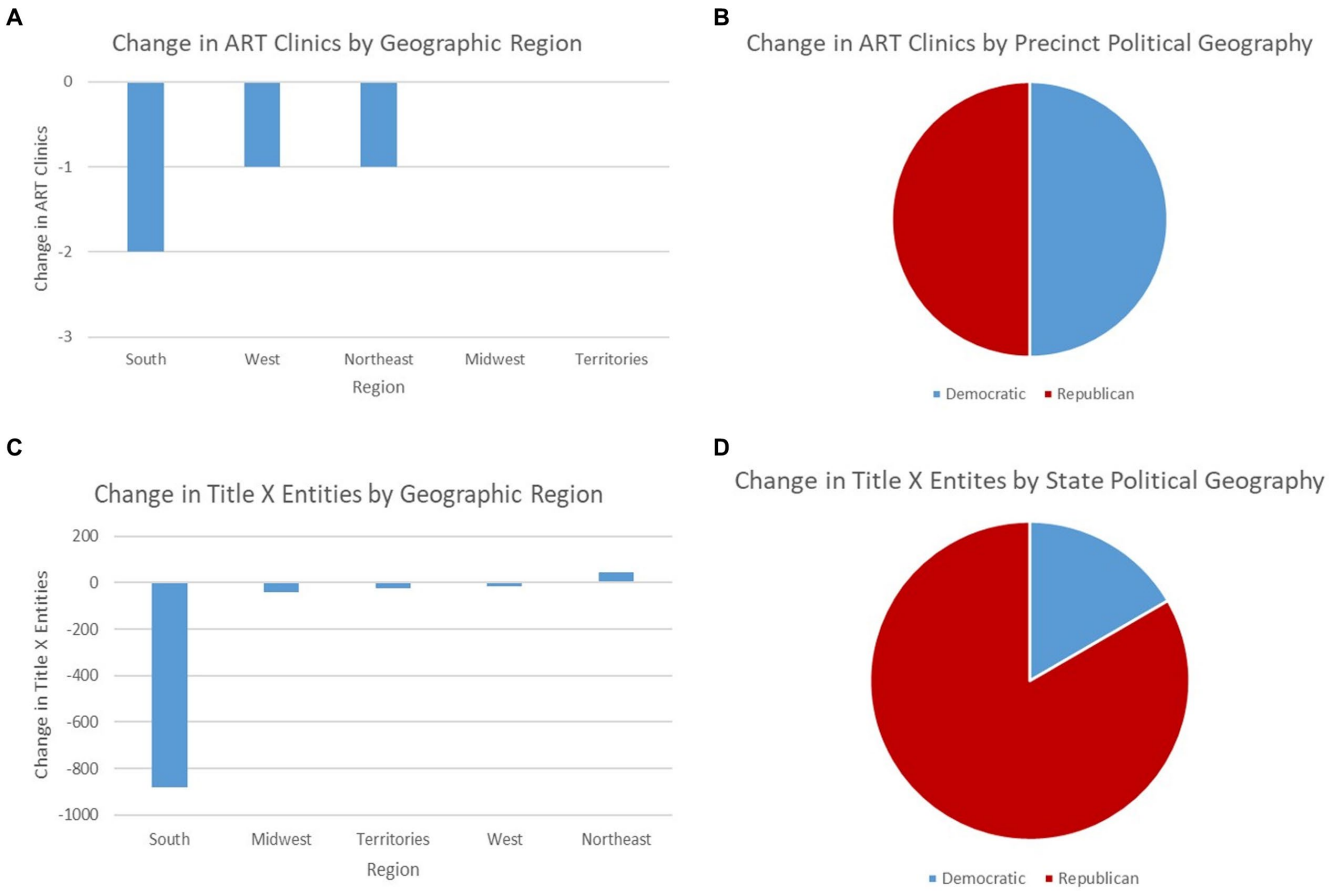
Pre-post *Roe* changes in ART clinics and Title X entities from the overturn to date (February 2023) by census region and political geography. **(A)** Change in ART clinics by census region. **(B)** Change in ART clinics by precinct political geography. **(C)** Change in Title X entities by census region. **(D)** Change in Title X entities by state political geography. **(A)** The number of closed ART clinics by census region from the overturn of *Roe* to date. All 495 ART clinics were extracted from the HHS-CDC 2020 ART annual report (20), and their operation status were manually collected for each clinic in the report. **(B)** The division of closed ART clinics by political geography. Authors located the precinct of each closed ART clinic using zip code within the Precinct-Level Returns 2020 by Individual State from the MIT Election Data and Science Lab (26) and determined which political party held the majority in the 2020 US presidential election. **(C)** The change in Title X entities by census region from the overturn of *Roe* to date. Data were extracted from the HHS-OPA Title X family planning monthly directory (19). **(D)** The division of changed Title X entities by political geography. Authors identified the state of changed Title X entities from the HHS-OPA Title X family planning monthly directory (19) and determined which political party held the majority in the 2020 US presidential election.

From the overturn of *Roe* to date, four ART clinics have closed. Two were located in the South, one in the West, one in the Northeast, and none in the Midwest. Two were in precincts and states that voted Democratic in the 2020 presidential election, and two were in those that voted Republican (Figures 3A,B). Closure reasons varied, one because of practice cessation, one because of relocation, merger, or acquisition, one because of financial losses, and one with unspecified reasons. Public releases by institutions may not convey underlying reasons, such as operational risks under stricter laws. For example, the hospital system Integris Health closed the Bennett Fertility Institute in Oklahoma after 37 years of operation on December 31, 2022, citing “declining patient volumes and overall financial losses from increased expenses and contract labor costs” (27). Coincidentally, this decision was made after the Oklahoma abortion trigger law was passed in May 2022 (28). Anecdotal evidence from physicians at Bennett revealed that the sudden closure resulted in layoffs, treatment discontinuation, and patient anxiety about the safety of frozen sperms, oocytes, and embryos (27).

### Title X entities before-after overturn

Title X entities fell from 5,491 before the overturn to 4,571 to date (19), a 16.75% decrease nationwide. State-level group means suggested a significant average loss of 18 Title X entities per state (95% CI, −7.4829 to −27.9020; *p* < 0.001). The largest loss and gain in Title X entities were in VA (126 lost) and OR (82 gained). Moreover, state-level changes in Title X entities and abortions performed were statistically significantly correlated [*r*(49) = 0.304; *p* = 0.0298] and associated (*β* = 0.023; 95% CI, 0.003 to 0.044; Figure 2B): Reducing 100 abortions was associated with defunding two Title X entities.

### Characteristics of gained Title X entities after review (April 2021)

From the review of *Dobbs* to date, the US has experienced a net gain of 1,036 Title X entities, composed of a 1,956 net gain from the review to the overturn and a 920 net loss from the overturn to date. This gain from the review to the overturn was mainly attributed to the November 2021 Biden-Harris Title X rule (29), consistent with the observed increase in early 2022 (Figure 1B). The 2021 Biden-Harris rule repealed the March 2019 Trump gag rule (30). The 2019 gag rule prohibited Title X entities from abortion referrals, disqualified those that practiced abortion, and required entities to encourage family participation in family planning decisions, among others (31). These requirements discouraged and disqualified entities from participating in Title X (32), consistent with the observed decrease in 2019 (Figure 1B).

Conversely, the 2021 Title X rule “remove[d] restrictions on nondirective options counseling and referrals for abortion services and eliminate[d] requirements for strict physical and financial separation between abortion-related activities and Title X project activities, thereby reversing the negative public health consequences of the 2019 regulations.” It also required entities to supply comprehensive family planning options to meet patient demands. These requirements and HHS implementations, such as $256.6 million in grant funding in March 2022 (33), foster the growth of Title X entities, consistent with an observed increase from 4,258 in 2021 to 5,491 in May 2022 (Figure 1B).

Among the net gain of 1,956 entities from the review to the overturn, 875 were gained in the South (44.73%), 458 in the West (23.42%), 414 in the Northeast (21.17%), 186 in the Midwest (9.51%), and 23 in US territories (1.18%). Moreover, 1,178 entities were gained in states that voted Democratic in the 2020 presidential election (60.94%) and 755 in states that voted Republican (39.06%).

### Characteristics of lost Title X entities after overturn (May 2022)

Conversely, from the overturn to date, the US has witnessed a net loss of 920 Title X entities, among which 880 were lost in the South (−95.65%), 43 in the Midwest (−4.67%), 23 in US territories (−2.50%), 16 in the West (−1.74%), and 42 were gained in the Northeast (4.57%). Moreover, 748 out of the 920 lost entities were in states that voted Republican in the 2020 presidential election (83.39%), disproportionately higher than the 49.02% of states that voted Republican and the 42.52% of US population residing in these states.

The federal judicial overturn of *Roe* resulted in the nationwide, state-level loss of Title X entities, likely through the mechanism of state abortion trigger laws taking effect immediately after the overturn as state-level barriers. Indeed, trigger laws in six states punish providers that “assist,” “abet,” or “employ any means to procure” abortion even out of the state; trigger laws in ten states criminalize and even felonize persons who attempt to or perform abortion (5). Such state trigger laws and their implementations did not comply with the 2021 Title X rule, resulting in the federal government discontinuing Title X funding to entities in these states (34).

## Worsening challenges and future directions

Evidence uncovers four worsening challenges. Federal de-subsidization of Title X entities due to state-level barriers inevitably shifts contraceptive and preventive service costs to patients, especially low-income men and women in Southern and Western Republican-leaning states. Historically, fewer Title X entities resulted in “contraception deserts” (21), while losing Title X funding led entities to shift service costs to patients and was criticized by clinicians and administrators (35). Similarly, women’s health center closures after 2011 state budget cuts increased the distance to the nearest center and decreased preventive care utilization among women of lower educational attainment (36). The post-*Roe* plunge in Title X entities can exacerbate limited contraceptive and preventive services access.

The descending trend since the mid-2010s and the recent closure of ART clinics and its positive association with the recent fall in abortions in Texas, Oklahoma, Arizona, and New York suggest decreasing patient access to fertility services in these states. Past closure of ART clinics was associated with service delays and cancelations (37). Patients with access often still need multiple ART cycles due to average low success rates. In 2020, only 79,942 births were produced out of the 326,741 total cycles performed, leaving a national success rate of 24.5% (20). Current closures can further exacerbate the overall decline in fertility (38).

Refusal of physicians to provide requested IVF treatment to socially infertile and fertile patients who prefer ART has historically been criticized. LGBTQ patients and single parents by choice have challenged ART clinics and state statutes for discrimination harmful to their reproductive health, such as in *Benitez v. North Coast Women’s Care Medical Group (2008)* (39) and *Krupa v. The New Jersey State Health Benefits Commission (2018)* (40). The declining trend in and recent closure of ART clinics can aggravate longstanding income, gender, sexual orientation, and marital status inequities in accessing fertility services for diverse patient populations.

The fourth major challenge is a lack of disaggregated data to further quantify the magnitude of post-*Roe* impacts on non-abortion reproductive care demand, identify causal connections, and increase sample size at the county level, in addition to the supply trends and associations at the nationwide state level found in this article. National surveillance data on ART and abortion clinics have at least 2-year time lags (20), and the CDC only receives aggregate voluntary reports of the latter from state health agencies (23). The National Survey of Family Growth, last reported in 2019, has a small sample size of infertile individuals among married and cohabiting women only, excluding others, such as men and single and homosexual women. Administrative data limitations hinder the ability to estimate post-*Roe* impacts timely. Future efforts in data collection, causal inference, funding, and provider support are urgently needed to inform policy and protect non-abortion reproductive care access for diverse patient populations.

## Conclusion

Administrative and manually-collected data have shown early national trends of decreases in ART clinics and Title X entities after the US Supreme Court accepted to review *Dobbs* and overturned *Roe*. Data, funding, and provider support should be ensured to inform policy and protect a broad spectrum of reproductive services access needed by diverse populations, including men and women, low-income individuals, single parents by choice, and biologically and socially infertile patients.

## Author contributions

JZ: Conceptualization, Funding acquisition, Investigation, Methodology, Project administration, Resources, Supervision, Validation, Visualization, Writing – original draft, Writing – review & editing, AZ: Data curation, Formal analysis, Investigation, Software, Visualization, Writing – review & editing. SP: Conceptualization, Writing – review & editing. TQ: Conceptualization, Writing – review & editing. JC: Resources, Writing – review & editing. PH: Conceptualization, Resources, Writing – review & editing.
