# Supplementary material for: Early national trends in non-abortion reproductive care access after Roe
Source: Front Public Health. 2024 Mar 8;12:1309068. doi: 10.3389/fpubh.2024.1309068 (PMC10957616; doi:10.3389/fpubh.2024.1309068)
Supplement: Supplementary file 1 [file Data_Sheet_1.docx]

References used for ART clinic closure

1. California Secretary of State. Certificate of Election to Wind Up and Dissolve: Eden Fertility Center of Newport Beach, PC. Sacramento, CA: State of California; 24 July 2020. 1 p. Filing No.: D1581707.
2. California Secretary of State. Certificate of Dissolution: Eden Fertility Center of Newport Beach, PC. Sacramento, CA: State of California;4 March 2022. 1 p. Filing No. D1775939.
3. Georgia Secretary of State. Certificate of Merger: Reproductive Biology Associates, LLC and Aspire Fertility Institute, LLC. Atlanta, GA: State of Georgia; 5 October 2020. 3 p. Filing No.: 16089341.
4. Abae M. Notice of Practice Closure. *Fertility & Genetics*, https://web.archive.org/web/20201001231133/https://www.fertilityandgenetics.com/ (2020, accessed January 29, 2024).
5. Bellingham IVF. Bellingham IVF & Infertility Care, https://web.archive.org/web/20210927013147/https://www.bellinghamivf.com/ (2021, accessed January 29, 2024).
6. Arizona Reproductive Institute. Arizona Reproductive Institute. *Facebook*, https://www.facebook.com/ArizonaReproductiveInstitute/posts/pfbid0K93oxsAvvoRQt38k1qUwX2d9r5br7CeqKdFPm9rYchS7ewYBiYToneZpJuti36Rpl (2022, accessed January 29, 2024).
7. Alta Bates IVF. Alta Bates IVF Program, https://www.abivf.com/#popup (2022, accessed January 29, 2024).
8. Bass K, Padilla S. Dr. Bass and Dr. Padilla are Retired. *Fertility Center of Maryland*, https://web.archive.org/web/20220306174210/https://fertilitycentermd.com/ (2022, accessed January 29, 2024).
9. New York Fertility Institute. New York Fertility Institute, https://nyfertility.org/ (2022, accessed January 29, 2024).
10. 1. INTEGRIS Health. INTEGRIS Bennett Fertility Institute Closing Dec. 31, 2022. *INTEGRIS Health*, https://integrisok.com/landing/bennett-fertility-institute-closing (2022, accessed January 29, 2024).
11. Women’s Health Texas San Antonio. Women’s Health Texas San Antonio. *Facebook*, https://www.facebook.com/WomensHealthTxHardyOak/posts/pfbid0onZvUNbJRb8c8oXfYTmptGUXBGDkocARgwa3JQS1bkU2XgZiPRLTAA4NMbyCVnGpl (2022, accessed January 29, 2024).
12. The Heard Institute. Heard Institute Practice Information. *The Heard Institute*, https://web.archive.org/web/20230405192329/https://www.theheardinstitute.com/ (2023, accessed January 29, 2024).
13. King L. Thank You. *The Women’s Place*, https://www.thewomensplace.net/ (2022, accessed January 29, 2024).
